# The Effect of Deposited Dust on SCC and Crevice Corrosion of AISI 304L Stainless Steel in Saline Environment

**DOI:** 10.3390/ma14226834

**Published:** 2021-11-12

**Authors:** Chun-Ping Yeh, Kun-Chao Tsai, Jiunn-Yuan Huang

**Affiliations:** Institute of Nuclear Energy Research (INER), 1000 Wenhua Rd., Longtan District, Taoyuan City 32546, Taiwan; tsaijohn@iner.gov.tw (K.-C.T.); jyhuang@iner.gov.tw (J.-Y.H.)

**Keywords:** chloride concentration, relative humidity, crevice corrosion, stress corrosion cracking, dust, stainless steel

## Abstract

Crevice corrosion has become an important issue of the safety of AISI 304L austenitic stainless steel canister when exposed to the chloride environments located in coastal areas. Moreover, dust deposited on the canister surface may enhance the corrosion effect of 304L stainless steel. In this work, white emery was adopted to simulate the dust accumulated on the as-machined specimen surface. To investigate the effect of deposited white emery, chloride concentration, and relative humidity on the crevice corrosion behavior, an experiment was conducted on 304L stainless steel specimens at 45 °C with 45%, 55%, and 70% relative humidity (RH) for 7000 h. The surface features and crack morphology of the tested 304L stainless steel specimens were examined by SEM equipped with energy-dispersive spectrometry (EDS) and electron back scatter diffraction (EBSD). From the experimental results, a threshold RH for the stress corrosion cracking (SCC) initiation of AISI 304L austenitic stainless steel with different concentrations of chloride was proposed.

## 1. Introduction

The facilities of interim storage for storing spent nuclear fuel are located in coastal areas in Taiwan. The dry cask storage systems are expected to operate for approximately 40–60 years [1]. As a result, it is necessary to maintain the integrity of the stainless steel canisters for guaranteeing the safety of spent nuclear fuel storage. Canisters are stored in passively ventilated overpacks and accumulate dust containing chloride salts on their surface over a long period of time. When dust containing chloride salts is deposited on canister surfaces, it creates an environment where water accumulates and corrodes the region, which can lead to localized crevice corrosion. 

Stainless steel is susceptible to suffering from localized corrosion, particularly crevice corrosion occurring on surfaces [2]. It has been suggested that the crevice corrosion behavior of stainless steel is directly related to the passive film evolution process inside the crevice [3]. With the increase in chloride concentration, passive film becomes more unstable, which brings about more serious crevice corrosion [4]. In contrast to chloride ions, nitrate ions act as a passivating agent to inhibit pitting corrosion [5].

By trapping chloride deposits on the surface of canisters, crevice corrosion becomes one of the promoting factors of the so-called atmospherically-induced SCC (AISCC). The initiation of crevice corrosion is particularly dependent on aggressive Cl^−^ ions, crevice geometry, and temperature. In canister systems, crevice conditions occur where the canister contacts the support structure of a storage module [6].

In terms of austenitic stainless steel, it is subject to SCC, especially in an aggressive environment, such as stress corrosion cracking due to Cl^−^ ions [7,8,9]. An increased volume of corrosion products possibly creates localized stress at the local sites of crevices to induce the occurrence of the stress corrosion cracking. In addition, Tani et al. reported that crevice corrosion causes the initiation of SCC on the surfaces beneath sea salt particles [10].

The following mechanisms are usually used to explain the behavior of crevice corrosion: The mechanism of critical crevice solution potential drop [3], in which the oxygen depleted in the crevice leads to acidification of the crevice solution, which generates breakdown of the passive film and initiation of the crevice corrosion. This mechanism places emphasis on aggressive ions, such as chloride ions accumulated in the crevice, and, following depassivation, results from active dissolution of the base metal [11]. As for the mechanism of IR drop, when the value of the IR exceeds a critical threshold, crevice corrosion occurs by means of transiting the potential from the passive state to the active state. By increasing the corrosion current, aggressive ions (e.g., Cl^−^) facilitate crevice corrosion, which causes an increase in the IR value [12]. 

The areal density of the salt deposit and local environmental conditions determine the chloride concentration present on the canister surface. The Southwest Research Institute (SwRI) [13] conducted experiments at a chloride concentration of 0.1–10 g/m^2^ of synthetic sea salts deposited on 304, 304L, and 316L stainless steel U-bend specimens. Dong et al. investigated the SCC susceptibility of deposited MgCl_2_ on 316L stainless steel at 75 °C with a relative humidity of 70% for 480 h. They suggested that fine transgranular SCC (TGSCC) appeared from the side of the pits of the weld region [14]. Corrosion pits can also be connected to tensile stress. Higher tensile stress is prone to increasing the pitting density in the heat-affected zone (HAZ) [15,16]. Furthermore, the morphology of a crevice-like attack or pit could impact the susceptibility of the pit developing into a crack due to the morphology being able to affect the distribution of stress/strain [17]. Guo and coworkers reported atmospheric corrosion of 304 stainless steel under mixed salts of MgCl_2_ and NaCl at 21 °C and 45% RH. They suggested that dish-shaped pits can be observed in mixed salts solutions. Moreover, crevice corrosion occurs under NaCl crystals [18]. Shoji et al. proposed systematic research of the AISCC phenomenon in terms of chloride ions, relative humidity, and temperature, and illustrated its happening in 316L and 304L austenitic stainless steel. It was reported that MgCl_2_ is the constituent of sea salt responsible for enhancing low-temperature AISCC in AISI 316L and AISI 304L austenitic stainless steel [19] because of its low deliquescence point [20]. Scatigno et al. investigated the impact of salt on chloride-induced SCC in AISI 304L stainless steel at a 70% relative humidity and 90 °C. It was reported that AISCC was found in the environment with a chloride concentration of 1.7 g/m^2^ of MgCl_2_ [21]. Engelberg and coworkers provided an order of susceptibility for the occurrence of atmospherically induced SCC of 316L and 304L austenitic stainless steel. Moreover, the RH ranges over which AISCC took place at a chloride concentration of 25 g/m^2^ of MgCl_2_ and sea water were summarized, based on the work of Shoji and coworkers [22]. Prosek et al. suggested that the corrosivity of deposited chloride under specific exposure situations was in the descending order of: CaCl_2_ > MgCl_2_ > NaCl. Due to interaction of the given salt with the water vapor in the air, it was controlled by the chloride concentration on the surface [23]. Furthermore, the threshold of the chloride concentration for the SCC initiation of 304L steel after 10,000 h of testing at 45 °C was between 0.1 and 1 g/m^2^ [24]. Moreover, Ornek et al. reported that based on salt loading tests for the 304L stainless steel, the chloride concentration at the crack tips was saturated and, hence, the bulk concentration of chloride at the surface did not affect the crack propagation rates [25].

Masuda et al. presented the SCC phenomenon of AISI 304 steel with regard to the growth of a pit, surface potential distribution, and slip deformation. They conducted SCC tests at 343 K and a relative humidity of 28% with MgCl_2_ droplets deposited on specimens. Discrete cracks were usually found near the region of the crack tip [26]. Qiao et al. investigated the SCC phenomenon of 321 stainless steel in an MgCl_2_ solution. They reported that the interaction between the main crack and discontinuous microcracks may promote the effective stress intensity factor and enhance the coalescence of the crack, resulting in mechanical fracture of the ligaments between the cracks in stainless steel [27].

Dust accumulation is prone to being affected by the roughness of the material surface. Higher surface roughness insinuates the presence of deeper grooves on the material surface, where a higher rate of dust accumulation and a higher concentration of ions occur, causing early initiation of corrosion [28].

The aim of this research was to survey the behavior of the crevice corrosion of AISI 304L steel under the combined effect of accumulated dust, different chloride concentrations, and relative humidity, and the experimental results of this research are expected to contribute to evaluating the durability of 304L canisters located in coastal areas. 304L stainless steel specimens with as-machined surfaces were adopted in this work to accelerate the effect of crevice corrosion for dust accumulated on the specimens’ surfaces. Furthermore, this research offers greater knowledge of the initiation of stress corrosion cracking at sites of crevice corrosion.

## 2. Experimental Procedure

**Figure 1:**
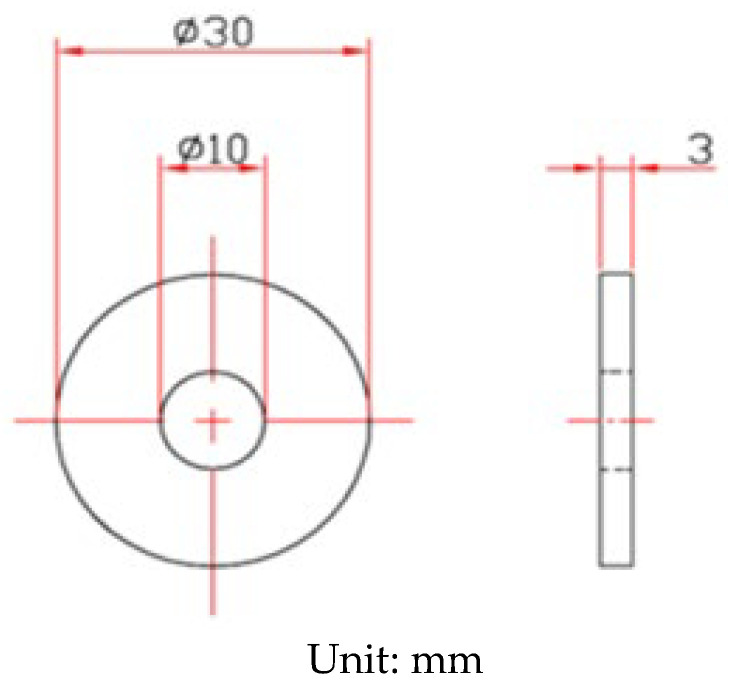
Dimensions of the specimen used for crevice corrosion test.

The shape and dimensions of the test specimens are presented in Figure 1. White emery was adopted in this work to simulate the dust accumulated on the as-machined specimen surface. The as-machined surface roughness (Ra) of the specimen was 4.9 μm. The chemical compositions of the AISI 304L steel used in the work are listed in Table 1. The test samples were manufactured from AISI 304L austenitic stainless steel, and white emery was made from alumina. Duplicate tests were conducted for each experimental condition.

**Figure 2:**
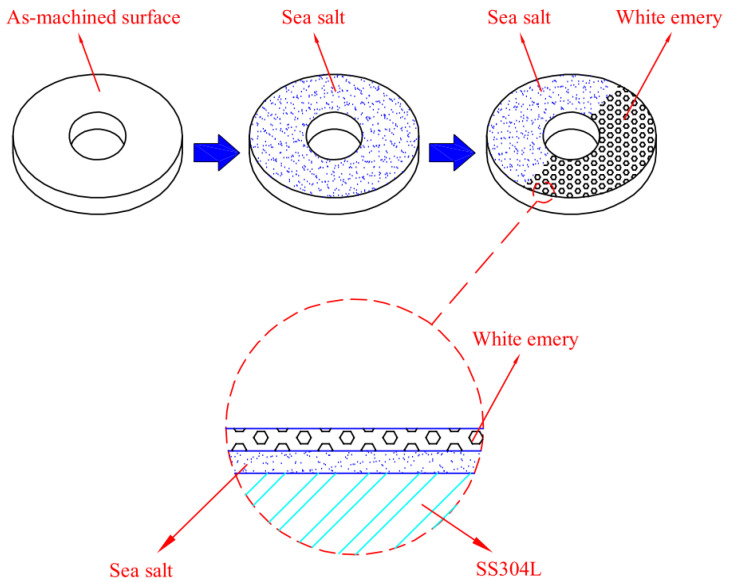
Schematic diagram of the specimen sprayed with synthetic sea water and white emery.

The chemical composition of sea salt used in the present study, based on ASTM D 1141-98 (13) Formula a, are listed in Table 2. A schematic diagram of the test specimen sprayed with synthetic sea water and white emery is presented in Figure 2. First of all, the samples were sprayed with synthetic sea water of 3.5 wt.% and subsequently dried on a hot plate at 65 °C for 20 min. After the specimens were dried, the mass change of specimens was measured to calculate the salt loadings in g/m^2^. The concentration of chloride for the samples was 0.1 g/m^2^ and 1 g/m^2^. Finally, 0.5 g of white emery was sprayed uniformly in a semicircle of the sample. The constant humidity and temperature chambers were maintained at 45 °C with 45%, 55%, and 70% RH for 7000 h. Microstructural examination was acquired through a proper metallographic preparation process of the samples. The samples were washed ultrasonically in deionized water and dried carefully, then mounted in resin. The mounted specimens were polished using a powder of aluminum oxide. The microstructures and the surface features of the specimens were characterized with an optical microscope (OM) and the scanning electron microscope (SEM). The compositions of the corrosion products were determined by the technique of energy-dispersive spectrometer (EDS). Furthermore, a scanning electron microscope equipped with an electron backscatter diffraction (EBSD) detector was used to inspect the stress corrosion cracking features of the specimens. Moreover, a kernel average misorientation (KAM) map and strain countering map for samples were acquired by inputting the electron back scatter diffraction map into the HKL-Channel 5 software for conducting data processing. The crack morphologies of the samples were examined by SEM.

## 3. Results and Discussion

### 3.1. Surface Morphology Analysis

**Figure 3:**
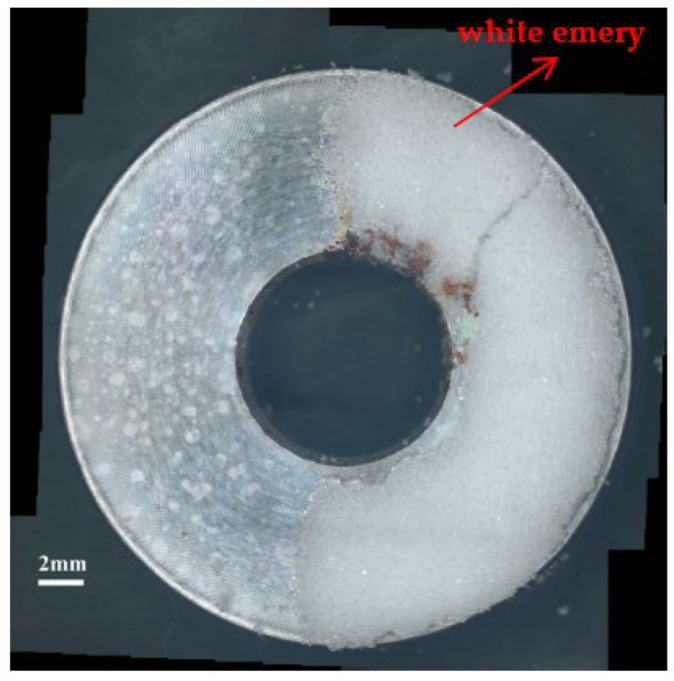
Macrographs of the specimens with a 1 g/m^2^ chloride concentration after 7000 h of testing a relative humidity (RH) of 70%.

**Figure 4:**
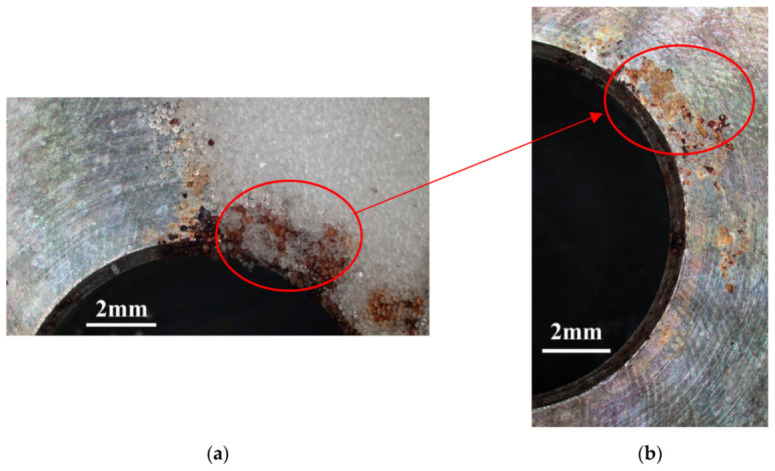
Macrographs of the corrosion regions of those samples with a chloride concentration of 1 g/m^2^ at RH = 70%: (**a**) Specimen before cleaning and (**b**) specimen after cleaning.

**Figure 5:**
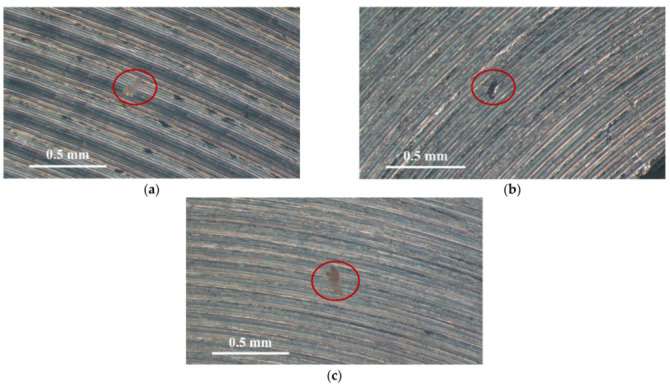
Macrographs of the corrosion regions of those samples with a 0.1 g/m^2^ chloride concentration at: (**a**) Relative humidity = 45%, (**b**) relative humidity = 55%, and (**c**) relative humidity = 70%.

**Figure 6:**
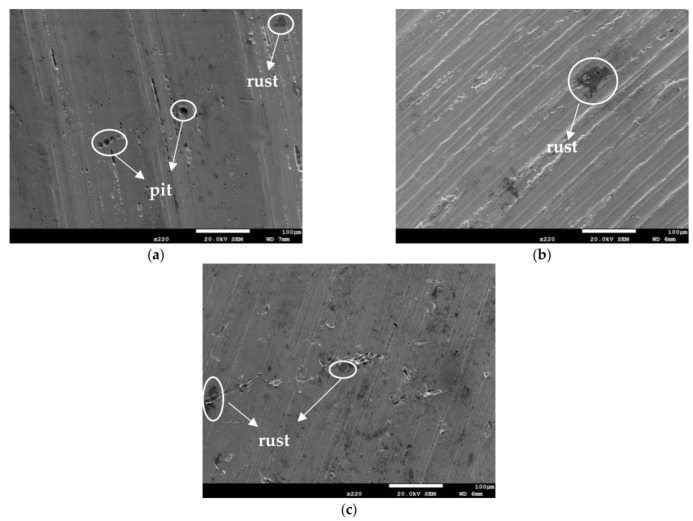
SEM micrographs of those specimens with a 0.1 g/m^2^ chloride concentration at: (**a**) Relative humidity = 45%, (**b**) relative humidity = 55%, and (**c**) relative humidity = 70%.

**Figure 7:**
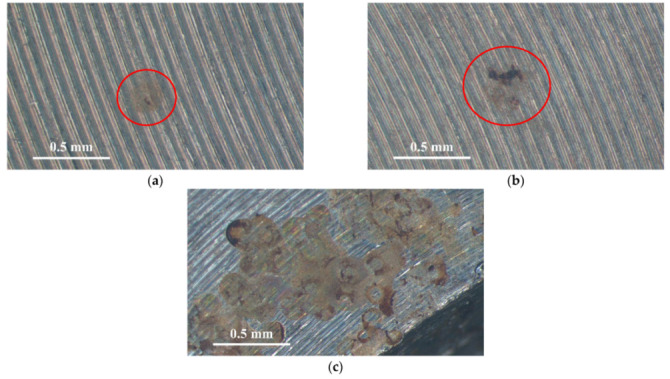
Macrographs of the corroded regions of those samples with a 1 g/m^2^ chloride concentration at: (**a**) Relative humidity = 45%, (**b**) relative humidity = 55%, and (**c**) relative humidity = 70%.

**Figure 8:**
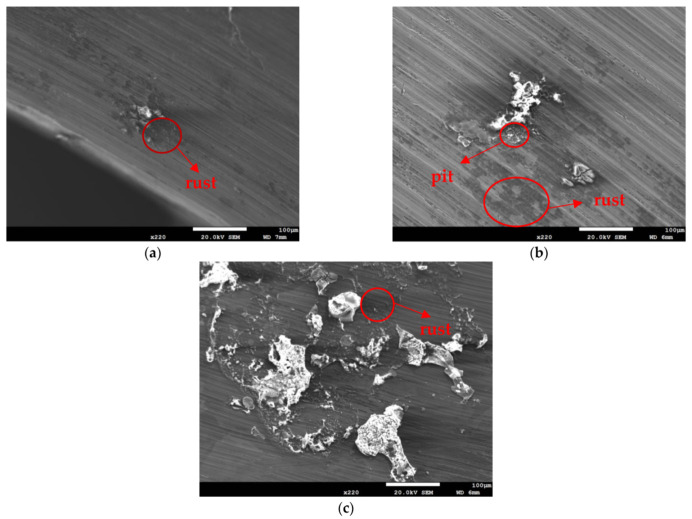
SEM micrographs of those samples with a chloride concentration of 1 g/m^2^ after 7000 h of testing at: (**a**) RH = 45%, (**b**) RH = 55%, and (**c**) RH = 70%.

Figure 3 and Figure 4 illustrate macrographs of the AISI 304L steel samples with a 1 g/m^2^ concentration of chloride after testing for 7000 h at a relative humidity of 70%. Figure 3 reveals that significant crevice corrosion occurred in corroded areas. Figure 4a illustrates that obvious crevice corrosion was only observed in the regions deposited with white emery, while Figure 4b demonstrates a clear image of the crevice corrosion after the specimen was cleaned. Figure 5 reveals macrographs of the AISI 304L stainless steel samples with a 0.1 g/m^2^ chloride concentration at three different relative humidity levels. A trace of crevice corrosion was discerned with the specimens. The rusted areas of the specimens at a relative humidity of 70% seem to be larger than the specimens at relative humidities of 45% and 55%. Figure 6 shows SEM micrographs of the samples deposited with a chloride concentration of 0.1 g/m^2^ at various levels of relative humidity for 7000 h. The rust observed on the specimens presents proof of crevice corrosion, which was induced by chloride. The morphologic features of the crevice corrosion changed with RH. Figure 6a reveals some small corrosion pits on the specimens. Furthermore, there are some rust spots on the specimens shown in Figure 6b,c, with shallow corrosion existing beneath the rust spots. Figure 6c shows that the specimens subjected to a relative humidity of 70% present slightly more corrosion than those exposed to relative humidities of 45% and 55%, as shown in Figure 6a,b. Figure 7 demonstrates macrographs of the AISI 304L steel samples deposited with a chloride concentration of 1 g/m^2^ at three different relative humidity levels. Figure 7a–c demonstrates that there is no obvious dissimilarity in the corroded regions on the samples at relative humidities of 45% and 55%. However, the corroded areas increased significantly at a relative humidity of 70%. In addition, the samples with a chloride concentration of 1 g/m^2^, shown in Figure 7, reveal more severe corrosion at all three RH levels than that with a chloride concentration of 0.1 g/m^2^, as illustrated in Figure 5. Figure 8 reveals SEM micrographs of the samples deposited with a chloride concentration of 1 g/m^2^ at various levels of RH for 7000 h. As for those samples deposited with a chloride concentration of 0.1 g/m^2^, the rust on the samples with a chloride concentration of 1 g/m^2^ is proof of crevice corrosion, which was induced by chloride. Figure 8a–c illustrate several rust spots that occurred on the specimen and shallow corrosion beneath the rust spots. The samples subjected to a relative humidity of 70% (Figure 8c) reveal more serious corrosion than those exposed to relative humidities of 45% and 55% (Figure 8a). Moreover, those samples with a chloride concentration of 1 g/m^2^, as illustrated in Figure 8, reveal more serious corrosion at all three RH levels than those with a chloride concentration of 0.1 g/m^2^, as shown in Figure 6.

### 3.2. EDS and EBSD Analyses

**Figure 9:**
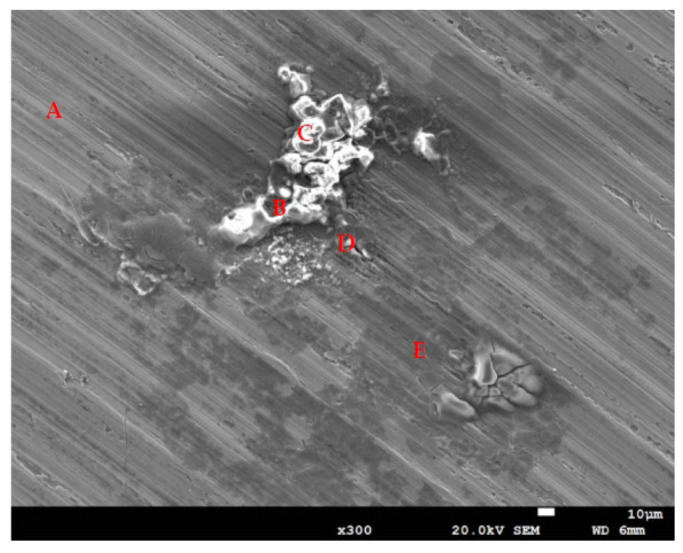
Energy dispersive X-ray spectrometry (EDS) analysis of the corroded regions on those specimens with a 1 g/m^2^ chloride deposit after testing at 55% relative humidity for 7000 h.

Figure 9 depicts EDS examinations of the corroded regions on test specimens deposited with a chloride concentration of 1 g/m^2^ at 55% RH. The results of the EDS analysis of the corroded regions are shown in Table 3, revealing that the sulfur and chlorine contents of Point A, which is the matrix of stainless steel, were so low that they could not be detected. Points B, C, and D, located in the corrosion products, were inspected to have higher contents of sulfur and chlorine, and the rust at Point E contained trace amounts of sulfur and chlorine. Furthermore, Points B, C, D, and E had higher contents of sulfur in comparison to Point A, which is probably explained by the sulfates contained in the synthetized sea water.

Cl^−^ ions are indispensable for the initiation of crevice corrosion. The CCS mechanism, owes the initiation of crevice corrosion to the accumulation of the aggressive ions, particularly Cl^−^ in the crevice, brings about the appearance of highly aggressive localized corrosion that destroys the passive film of stainless steel [29]. Point B has a higher content of iron but a lower content of oxygen compared to Points C and D, which indicates that the oxide product was peeled off to expose the metal surface. Moreover, Point E, located in the rust region, has a higher Fe content but lower Cl and O contents compared to Points B, C, and D.

**Figure 10:**
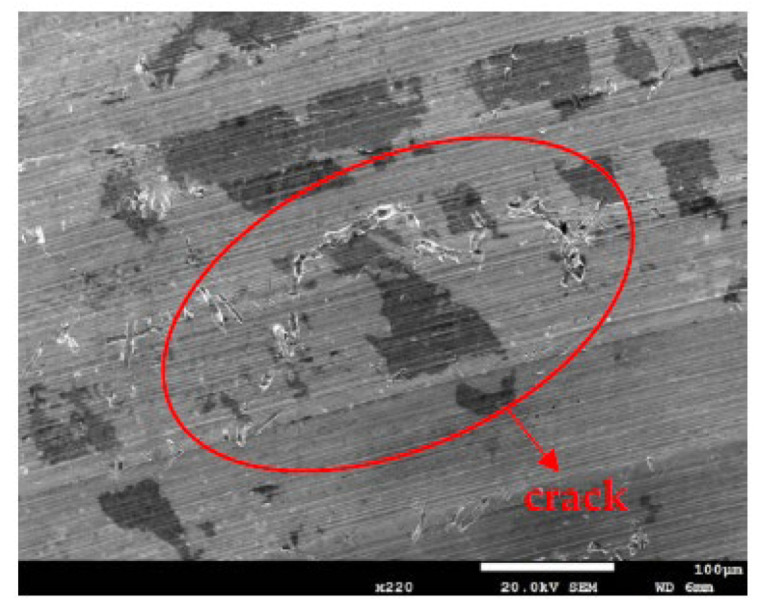
SEM morphology of the SCC in those samples with 0.1 g/m^2^ of chloride deposited after 7000 h of testing at RH = 70%.

**Figure 11:**
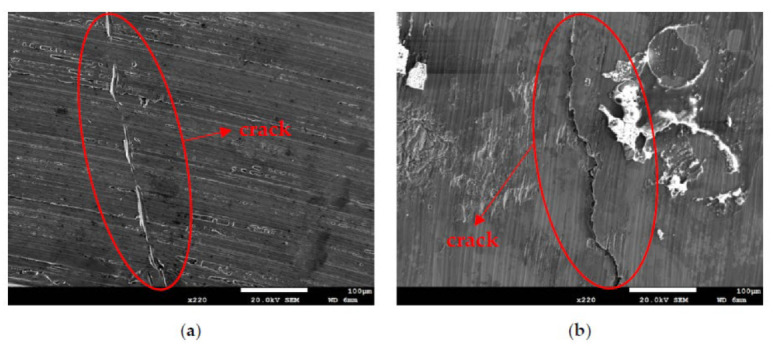
SEM morphology of the SCC in those samples with a chloride concentration of 1 g/m^2^ after 7000 h of testing at: (**a**) RH = 55% and (**b**) RH = 70%.

Figure 10 and Figure 11 are SEM micrographs demonstrating the morphology of the SCC of the samples deposited with chloride concentrations of 0.1 g/m^2^ and 1 g/m^2^ at various RH levels, respectively. For samples deposited with a 0.1 g/m^2^ chloride concentration, no SCC cracks were observed on those specimens exposed to the 45% and 55% relative humidities, as shown in Figure 6a,b, whereas Figure 10 demonstrates discontinuous SCC cracks on those specimens tested at a 70% relative humidity. For samples deposited with a 1 g/m^2^ chloride concentration, no SCC cracks were observed on those specimens exposed to a 45% relative humidity, as exemplified in Figure 8a. Meanwhile, Figure 11a shows discrete SCC cracks on those samples exposed to a 55% RH, whereas Figure 11b reveals continuous SCC cracks on the specimens tested at a 70% relative humidity. Shoji et al. demonstrated that MgCl_2_ is the sea salt constituent responsible for promoting low-temperature atmospherically induced SCC in AISI 316L and 304L steel. They inspected the phenomenon of atmospherically induced SCC of samples deposited with a 25 g/m^2^ chloride concentration using sea water [19]. Prosek and coworkers investigated the phenomenon of atmospherically induced SCC of AISI 316L and 304 stainless steel with a 260 g/m^2^ chloride concentration acquired by MgCl_2_ droplets [23]. The maximum 1 g/m^2^ chloride concentration used in this research is much lower compared to that reported in the literature [19,22,23]. Figure 10 and Figure 11a demonstrate short and shallow cracks on those samples deposited with 0.1 g/m^2^ of chloride at a 70% RH and 1 g/m^2^ of chloride at a 55% RH, respectively, as opposed to Figure 11b, in which long and deep cracks appear on those specimens deposited with 1 g/m^2^ of chloride at a relative humidity of 70%. It is conjectured that microcracks nucleate discontinuously at favorable sites in the first place and, under some favorable situations, microcracks grow and then coalesce by means of breaking ligaments between them to connect into one main crack [26,27,28,30]. On the basis of this hypothesis, some of the shallow and short cracks present on those samples with a 0.1 g/m^2^ chloride concentration at a 70% RH and a 1 g/m^2^ chloride concentration at a 55% RH could be attributed to finite amounts of Cl^–^ ions transmitted to the crevice sites of the samples. As a result of the low concentration of chloride of the samples tested, it is essential to have higher RH environments to promote the transmission of adequate amounts of Cl to crevice sites for promoting crack nucleation. The Cl maps obtained by EDS mapping are proof of the aforementioned argument. There was no SCC at RH = 45% and 55% whereas specimens exposed at RH = 70%, the highest RH of the three, cracked with those specimens deposited with a chloride concentration of 0.1 g/m^2^, as shown in Figure 6a,b and Figure 10. This is probably because the chloride concentration of 0.1 g/m^2^ was so low that it could not initiate stress corrosion cracking in the specimens tested at lower levels of relative humidity (RH = 45% and 55%). Moreover, when the chloride concentration for the specimens was increased to 1 g/m^2^, there were still no SCC cracks with specimens exposed to RH = 45% whereas those exposed to RH = 55% and 70% cracked, as illustrated in Figure 8a and Figure 11a,b. This can be possibly interpreted as finite chloride transmitted into crevice sites at the lowest relative humidity (relative humidity = 45%) in the specimens deposited with 1 g/m^2^ of chloride. Therefore, the synergistic effect of the chloride concentration and relative humidity could account for the SCC initiation of the 304L stainless steel. From the experimental results of the work, it can be concluded that the relative humidity threshold for the SCC initiation of AISI 304L stainless steel with an as-machined surface deposited with a chloride concentration of 0.1 g/m^2^ at 45 °C is between 55% and 70% RH, whereas the relative humidity threshold for those specimens deposited with a chloride concentration of 1 g/m^2^ is between 45% and 55% RH. The increased volume of corrosion products possibly creates localized stress beneath white emery, which acts like a crevice former, leading to the occurrence of SCC. Furthermore, with the increase in relative humidity, the length of the cracks was observed to increase when comparing Figure 11a to Figure 11b.

**Figure 12:**
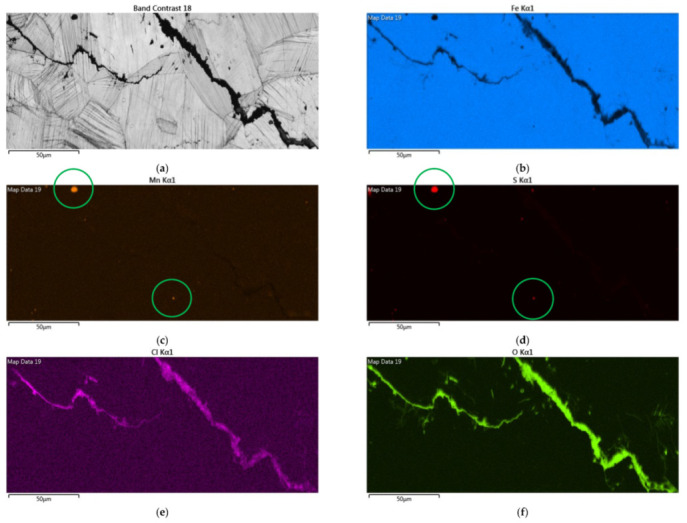
EDS mapping for the crack regions of those samples with a chloride concentration of 1 g/m^2^ tested for 7000 h at a relative humidity of 70%: (**a**) Band contrast image, (**b**) Fe mapping, (**c**) Mn mapping, (**d**) S mapping, (**e**) Cl mapping, and (**f**) O mapping.

Figure 12 demonstrates EDS mapping for the crack regions of those samples deposited with a 1 g/m^2^ chloride concentration tested at a 70% RH. The crack region is obviously enriched with chlorine and oxygen, as respectively demonstrated by Figure 12e,f, but depleted with iron, as manifested in Figure 12b. Figure 12c,d reveal enriched manganese and sulfur, respectively, on the center bottom and left top of the figure.

**Figure 13:**
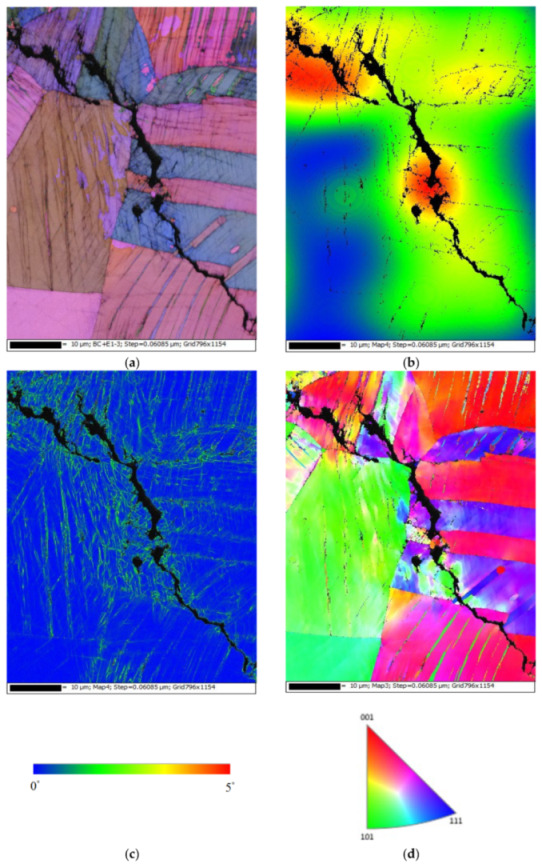
Electron back scatter diffraction (EBSD) maps for the crack region of the specimens deposited with a 1 g/m^2^ chloride concentration tested for 7000 h at 70% RH: (**a**) Euler map, (**b**) strain countering map, (**c**) kernel average misorientation (KAM) map, and (**d**) inverse pole figure (IPF) map.

Figure 13 demonstrates EBSD maps for the crack regions of those specimens deposited with 1 g/m^2^ of chloride at a 70% RH. Figure 13a,d present samples cracked by a transgranular stress corrosion cracking (TGSCC) mode, which is in good agreement with those findings on transgranular stress corrosion cracking of stainless steel induced by chlorides when the temperature was above 50 °C [10]. The strain countering map for the specimens tested (Figure 13b) illustrates that the stress is more concentrated around the crack regions. Moreover, the kernel average misorientation (KAM) map for the specimens tested (Figure 13c) demonstrates that the high plastic strain, located around the crack regions, is probably linked to the plastic strain at the crack tip.

## 4. Conclusions

In this work, the crevice corrosion behavior of 304L stainless steel with white emery deposited on the as-machined surface was inspected by testing the samples at 45 °C under a combination condition of 0.1 g/m^2^ or 1 g/m^2^ chloride concentration and a RH of 45%, 55%, or 70% RH. The conclusions of this work are given as below:

(1) No cracks were observed on the samples deposited with a 0.1 g/m^2^ chloride concentration at a 45% and 55% RH. However, discontinuous cracks were found on those samples exposed to a 70% relative humidity, which is the highest RH of the three. This could be accounted for by the fact that the chloride concentration of 0.1 g/m^2^ was so low that it could not initiate stress corrosion cracking in the specimens tested at lower relative humidity levels (45% and 55% RH).
(2) When the chloride concentration for the specimens was increased to 1 g/m^2^, there were still no SCC cracks on those specimens exposed to RH = 45% whereas those exposed to 55% and 70% RHs cracked. Furthermore, discrete SCC cracks were found on those specimens exposed to a 55% RH, whereas continuous SCC cracks were observed on those exposed to a relative humidity of 70%. This could be interpreted by finite chloride being transported into the crevice sites at a 45% RH for the specimens deposited with 1 g/m^2^. The observation that the crack region had obviously been enriched with chloride provides evidence to verify the aforementioned argument.
(3) The results suggest that the relative humidity threshold was between 55% and 70% RH for the SCC initiation of 304L stainless steel with an as-machined surface and a 0.1 g/m^2^ chloride concentration at 45 °C, whereas the relative humidity threshold decreased to between 45% and 55% RH when the chloride concentration for the specimens increased to 1 g/m^2^.
(4) The specimens with a chloride concentration of 1 g/m^2^ had obvious crevice corrosion only at those regions deposited with white emery.
(5) The 304L stainless steel specimens with an as-machined surface tested at 45 °C were cracked with the TGSCC mode, which was substantiated by the results of EBSD.

## Figures and Tables

**Table 1:** Chemical composition of the 304L stainless steel used in this study.

Element	C	S	Si	Ni	Cr	Mn	Fe
wt.%	0.017	0.0290	0.450	9.000	18.000	1.540	Bal.

**Table 2:** Chemical composition of the sea salt used in this study.

Composition	NaCl	Na_2_SO_4_	MgCl_2_	KCl	CaCl_2_	NaHCO_3_	KCl	KBr	SrCl_2_	H_3_BO_3_
wt.%	58.490	9.750	26.460	1.645	2.765	0.477	1.645	0.238	0.095	0.071

**Table 3:** Results of the EDS analysis of the corroded regions (wt.%).

Location	O	S	Cl	Mn	Cr	Ni	Fe
A	0.70	0.00	0.00	1.70	18.6	8.50	70.4
B	17.5	0.80	1.20	1.50	23.5	7.20	48.3
C	42.6	0.90	4.10	1.30	10.6	6.10	34.4
D	30.0	1.80	3.60	2.20	39.1	1.90	21.3
E	15.9	0.30	0.20	1.90	16.2	7.30	58.3

## Data Availability

Data sharing is not applicable to this article.
